# An Antibody Targeting Fibroblast Activation Protein Simultaneously Fused to Interleukin-2 and Tumor Necrosis Factor Selectively Localizes to Neoplastic Lesions

**DOI:** 10.3390/antib12020029

**Published:** 2023-04-14

**Authors:** Eleonora Prodi, Claudia Comacchio, Ettore Gilardoni, Cesare Di Nitto, Emanuele Puca, Dario Neri, Roberto De Luca

**Affiliations:** 1 Philochem AG, Libernstrasse 3, 8112 Otelfingen, Switzerland; 2 CiBIO (Department of Cellular, Computational and Integrative Biology), University of Trento, 38123 Trento, Italy; 3 Philogen Spa, 53100 Siena, Italy

**Keywords:** antibody engineering, fibroblast activation protein, antibody–cytokine fusion proteins, interleukin-2, tumor necrosis factor

## Abstract

The delivery of specific cytokine payloads to a neoplastic environment employing antibodies able to selectively accumulate at the tumor site represents an attractive strategy to stimulate an immune response to cancer. Whilst conventional antibody–cytokine fusions based on a single payload have shown potent anticancer activity, the concomitant delivery of two cytokine payloads may further improve the therapeutic outcome as the immune system typically adopts multiple signals to reinforce an antitumor strategy. We here describe a potency-matched dual-cytokine antibody fusion protein containing a tumor-targeting antibody fragment specific to human fibroblast activation protein (FAP), simultaneously linked to both interleukin-2 (IL2) and a tumor necrosis factor (TNF) mutant. The resulting fusion protein, termed IL2-7NP2-TNF^mut^, formed stable non-covalent trimers driven by the interaction of the tumor necrosis factor subunits. Both cytokine payloads retained their biological activity within the fusion protein, as shown by in vitro cellular assays. The tumor-targeting properties and the anticancer activity of IL2-7NP2-TNF^mut^ were investigated in vivo in immunocompromised mice bearing SKRC52 cells transduced with human FAP. The fusion protein preferentially localized to the cancer site and induced partial tumor retardation.

## 1. Introduction

The recruitment of immune cells at the tumor site and their proliferation to fight cancer effectively represents one of the primary objectives of contemporary anticancer research. There is a growing interest in cytokine-based therapeutics as the immune system uses cytokines to modulate the activity of leukocytes [1]. Certain recombinant cytokine products have obtained marketing approval for cancer treatment despite severe side effects, providing a therapeutic benefit to a proportion of cancer patients [2,3,4,5,6,7,8]. Antibody–cytokine fusion proteins (also called immunocytokines), capable of a preferential localization at the tumor site by recognition of an accessible tumor-associated antigen, have been shown to increase the therapeutic efficacy of the cytokine payload, helping spare normal tissue [9,10,11,12,13,14,15,16,17].

The immune system frequently adopts multiple signals to reinforce a response against a pathogen or a neoplasm. For example, the action of CD8+ cytotoxic T cells against the tumor, bearing suitable peptides presented on MHC class I (signal 1), is potentiated by co-stimulatory molecules (signal 2) and by cytokine release (signal 3) [18]. Often, the simultaneous presence of increased concentrations of certain cytokine combinations facilitates a more robust antitumor response [19,20,21] or determines a specific differentiation pathway for T cells [19]. For this reason, our group and other groups have previously described a new class of immunocytokine products, termed dual-cytokine–antibody fusion proteins, in which a tumor-targeting antibody moiety was equipped with two cytokine payloads capable of a synergistic action.

One of the first examples of a dual-cytokine–antibody fusion featured interleukin-12 (IL12) and interleukin-2 (IL2) as payloads [22]. The targeted delivery of IL15 and 4-1BBL has also been reported with the generation of a trifunctional antibody fusion protein against fibroblast activation protein (FAP), showing therapeutic efficacy in a mouse model of lung metastases [23]. Additional examples of dual-cytokine fusions described in the literature and tested in preclinical cancer models include those based on anti-CD30 + IL2/IL12, anti-CD38 + IL2/TRAIL, and anti-HER2 + IL12/IL2 or GM-CSF [24,25,26,27].

When fusing antibodies to multiple cytokine payloads, a large number of formats can be considered. It has been shown that certain large arrangements of antibody–cytokine fusions may suffer from suboptimal pharmacokinetic and tumor-targeting properties, possibly associated with an excessive molecular weight and/or suboptimal glycosylation [28]. Additionally, the fusion of two cytokine payloads with different potencies may compromise the therapeutic performance. For example, IL12 and TNF typically exhibit an IC50 in biological assays in the 10^−12^–10^−13^ M concentration range, whereas IL2 and interleukin-4 (IL4) are generally active at a 10-fold higher concentration [29,30,31,32,33]. We recently described the use of “potency-matched” antibody–cytokine fusion proteins, in which a single amino acid mutation was introduced to effectively depotentiate the most active cytokine. The first molecule developed following this strategy was IL2-F8-TNF^mut^, in which IL2 and an R108A mutant of human TNF were fused to the F8 antibody, specific to the alternatively spliced extra domain A of fibronectin (EDA). The fusion protein induced complete cancer remission in immunocompetent tumor-bearing mice. It could be administered in higher doses to rodents than similar fusion proteins based on wild-type cytokine payloads [33,34].

Fibroblast activation protein (FAP) is one of the most investigated tumor-associated antigens expressed in the stroma of most tumor types whilst being almost undetectable in normal tissue [35,36,37,38,39,40,41,42]. High-affinity small organic FAP ligands equipped with radionuclides are routinely used for cancer imaging and tumor-therapy applications [43,44]. We recently described a fully human anti-FAP monoclonal antibody (7NP2) isolated from phage display libraries and submitted to a mutagenesis-based affinity maturation procedure. The antibody displayed excellent tumor-homing properties, suggesting that it could conveniently be used as a modular building block to generate anticancer therapeutics [45].

In this work, we report the production and characterization of a novel immunocytokine named IL2-7NP2-TNF^mut^. The protein was biologically active in vitro and localized to FAP-positive lesions. The therapeutic activity of IL2-7NP2-TNF^mut^ was compared with IL2-F8-TNF^mut^ in immunocompromised tumor-bearing mice, in which FAP was expressed on tumor cells whereas EDA was present in the tumor stroma. Unlike IL2-F8-TNF^mut^, IL2-7NP2-TNF^mut^ could not induce tumor necrosis, and only a modest tumor growth retardation was observed. Based on these data, target antigen localization (stromal or cellular) may be a fundamental factor that strongly influences the therapeutic success of this class of immunocytokine products.

## 2. Materials and Methods

### 2.1. Cell Lines

All the cell lines used in this study were obtained from 2018 to 2021, expanded, and preserved in aliquots stored in liquid nitrogen. The CTLL2 cell line was procured from ATCC, whilst the SKRC52 human renal cell carcinoma cell line was kindly provided by Professor E Oosterwijk from Radboud University Nijmegen Medical Center in The Netherlands. The SKRC52-hFAP cells were prepared following a previously described method [46]. All the cell lines were maintained in an RPMI medium supplemented with 10% fetal calf serum (FCS) and 1% antibiotic-antimycotic (AA), according to the supplier’s instructions, and were cultured for a maximum of 10 passages. Before shipment, the cell bank (ATCC) conducted a series of tests to authenticate the cell lines, which included assessing the post-freeze viability, growth characteristics, and morphology, as well as conducting tests for mycoplasma contamination, isoenzyme activity, and sterility.

### 2.2. Cloning, Expression, and Protein Purification

The gene encoding for IL2-7NP2-TNF^mut^ contained the antibody targeting human FAP in the ScFv format [45] fused at the N-terminus to human IL2 and the C-terminus to a mutated version of human TNFα [33]. The full sequence is reported in the supplementary material (Supplementary Figure S1). The genes were amplified, assembled, and inserted into the mammalian expression vector pcDNA3.1(+) (Invitrogen, Waltham, MA, USA) using *NheI/NotI* restriction enzymes. To produce the fusion proteins, we employed transient gene expression (TGE) in CHO-S cells following these steps: for each 1 mL of production, 4 × 10^6^ CHO-S cells grown in the suspension were centrifuged and then resuspended in 1 mL ProCHO4 (Lonza, Basel, Switzerland). Next, we gently mixed 0.75–0.9 µg of plasmid DNA and 2.5 µg of polyethyleneimine (PEI; 1 mg/mL solution in water, pH 7.0) per million cells with the cells. The transfected cultures were then incubated in a shaking incubator at 31 °C and 5% CO_2_ for 6 days. The resulting fusion proteins were purified from the cell culture medium by Protein A affinity chromatography, exploiting the VH properties of the 7NP2 antibody, and dialyzed against phosphate-buffered saline (PBS) with a pH of 7.4.

### 2.3. Biochemical Protein Characterization

To analyze the purified proteins, we subjected them to size-exclusion chromatography using a Superdex 200 Increase 10/300 GL column on an ÄKTA FPLC system (Cytiva, Marlborough, MA, USA). We also performed SDS-PAGE using 4–12% Bis-Tris gels (Invitrogen) under both reducing and non-reducing conditions. For differential scanning fluorimetry, we diluted the protein samples to 1 μM in 40 μL PBS and placed them in PCR tubes; we then added 5× SYPRO Orange (Invitrogen, stock 5000×) to the samples before the analysis. The assay was performed in triplicate, with the temperature range spanning from 25 °C to 95 °C and a scan rate of 1 °C/min. We used an Applied Biosystems StepOnePlus RT-PCR instrument and analyzed the data using Protein Thermal ShiftTM Software version 1.3 (Thermo Fisher, Waltham, MA, USA), computing the temperature derivative of the melting curve.

### 2.4. Affinity Measurements

For the Enzyme-Linked Immunosorbent Assay (ELISA), a Nunc^TM^ MicroWell^TM^ 96-Well Flat-Bottom Microplate (Thermo Scientific, Waltham, MA, USA) was coated with 0.1 µM human FAP antigen overnight at 4 °C in 50 mM HEPES, 100 mM NaCl with a pH of 7.4. Subsequently, the wells were blocked with 200 μL 10% milk-PBS and washed 3 times with PBS. Serial dilutions of IL2-7NP2-TNF^mut^ in 2% milk-PBS were titrated in the plate and incubated for 4 h at RT. The supernatant was discarded and the wells were washed with PBS before adding the Protein A-HRP detection antibody (1:1000, 100 µL/well, Invitrogen) in 2% milk-PBS and then incubated for 1 h at RT. Finally, the wells were rewashed 3 times with PBS-Tween (0.1% Tween20) and PBS, and 60 µL of the HRP substrate (BM Blue POD, Sigma-Aldrich, St. Louis, MI, USA) was added, and the reaction was quenched by 1 M H_2_SO_4_ (30 μL/well). Absorbance levels were measured on a Tecan Spark^®^ Multimode Microplate at 620 nm and 450 nm. Experiments were performed in triplicate.

### 2.5. In Vitro Biological Activities

The biological activity of the two cytokine payloads of IL2-7NP2-TNF^mut^ was evaluated by proliferation and killing assays. For the IL2 bioactivity, CTLL2 cells were seeded at 25,000 cells per well in 96-well plates with a titrated concentration of the fusion protein. After 48 h of incubation at 37 °C with 5% CO_2_, 20 µL of Cell Titer Aqueous One Solution (Promega, Madison, WI, USA) was added to the wells and the cell proliferation was measured by absorbance at 490 nm vs. 630 nm. The results were expressed as the percentage of cell viability compared with the untreated cells. A killing assay determined the biological activity of TNF on SKRC52 wt and SKRC52-hFAP cells. The cells were plated at 20,000 cells per well in two separate 96-well plates. The media were supplemented with actinomycin D (2 µg/mL, Sigma-Aldrich) and titrations of either recombinant human TNF wt or the fusion protein were added. After 24 h of incubation at 37 °C and 5% CO_2_, the cell viability was determined as the percentage of the cell viability compared with the untreated cells (negative control).

### 2.6. Flow Cytometry

SKRC52-hFAP cells were detached with 50 mM EDTA, pH 8.0, in PBS for 10 min at 37 °C. A total of 10 mL of RPMI was used for neutralization and the cells were centrifuged for 5 min at 900 rpm. A wash in 50 mL of a FACS buffer (0.5% BSA, 2 mM EDTA in PBS) was performed and the cells were resuspended at a final concentration of 5 × 10^6^ cells/mL. The cells were passed through a strainer to dissolve clumps and seeded at 500,000 cells of 100 µL into a 96-well U-bottom plate. The cells were incubated for 30 min on ice and then the plate was centrifuged for 3 min at 1500 rpm. Next, a titration of IL2-7NP2-TNF^mut^ primary antibody was added to the cells in 100 µL of the FACS buffer and incubated for 1 h on ice. Washing from the primary antibody was performed with 100 µL of the FACS buffer, then rat anti-IL2 (eBioscience, Waltham, MA, United States, catalog: 14-7029-85) and anti-rat AlexaFluor488 (Invitrogen A21208) were used for the detection of IL2-7NP2-TNF^mut^. Zombie NIR (Biolegend, San Diego, CA, USA) was used as live/dead staining and the data were acquired using a CytoFLEX cytometer (Beckman Coulter, Pasadena, CA, USA). The images were analyzed with FlowJo. The gating strategy is reported in the supplementary material (Supplementary Figure S2).

### 2.7. Immunofluorescence Studies

The FAP and EDA expressions were confirmed on 8 µm cryostat sections of SKRC52-hFAP fixed in ice-cold acetone. The primary antibodies utilized were 7NP2 IgG1, F8 IgG2a, KSF IgG4 (at 5 µg/mL) (the KSF antibody is specific for an irrelevant antigen), and rat anti-mouse CD31 for the staining of blood vessels (R&D AF3628). Detection was performed with goat anti-mouse Alexa 594 (Invitrogen A11005), goat anti-human Alexa 488 (Invitrogen A11013), and donkey anti-rat Alexa 594 (Invitrogen A11058). The tumor antigen expression was confirmed utilizing IL2-7NP2-TNF^mut^, IL2-F8-TNF^mut^, and IL2-KSF-TNF^mut^. In this case, the detection was performed with rat anti-IL2 (eBioscience 14-7029-85) and anti-rat AlexaFluor488 (Invitrogen A21208). For vascular staining, goat anti-mouse CD31 (R&D, Minneapolis, MI, USA, catalog: AF3628) and anti-goat AlexaFluor594 (Invitrogen A11058) antibodies were used. The cell nuclei were stained with DAPI (Invitrogen; D1306). Finally, the slides were mounted with a fluorescent mounting medium (Dako, Santa Clara, CA, USA) and analyzed with a wide-field Leica TIRF microscope using Leica LAS X Life Science Microscope Software (v 1.4.4, Wetzlar, Germany).

### 2.8. Mice and Tumor Models

This study involved the use of 30 female BALB/c nude mice, which were 8 weeks old and weighed an average of 20 g. The mice were purchased from Janvier (Route du Genest, 53940 Le Genest-Saint-Isle, France) and were raised in a controlled environment that was free from pathogens with a relative humidity of 40% to 60% and maintained at a temperature of 18 °C to 26 °C with a 12 h light/dark cycle. The guidelines of GV-SOLAS and FELASA were followed. The mice were housed in groups of 5 or fewer per cage; if necessary, single housing was provided in another cage. Blinding of the experimental groups was not performed. The mice were assigned to experimental groups based on their tumor volume and their tumor growth was monitored daily by measuring the tumor volume with a caliper (volume = length × width × 0.52). The subcutaneous implantation of 6 × 10^6^ SKRC52-hFAP cells was performed on the flank of each mouse.

### 2.9. Immunofluorescence-Based Biodistribution

For the immunofluorescence-based biodistribution study, the tumor-bearing mice were intravenously injected once with 60 µg/mouse of IL2-7NP2-TNF^mut^, IL2-F8-TNF^mut^, and IL2-KSF-TNF^mut^ diluted in Ringerfundin (B. Braun, Bethlehem, PA, USA). At 24 h post-administration, the mice were sacrificed and the tumors and organs were collected in a cryo-embedding medium (NEG-50 Thermo Fisher) for the further analysis. The cryostat sections were stained and detected, as previously described.

### 2.10. Mice Therapy Studies

When the tumors reached a suitable volume (approximately 100 mm^3^), the mice were injected 3 times into the lateral tail vein every 24 h with 30 µg of IL2-7NP2-TNF^mut^, IL2-F8-TNF^mut^, or IL2-KSF-TNF^mut^. Mice with subcutaneous tumors that showed ulceration or were larger than 15 mm in length or width were euthanized as the experimental endpoint. A loss of body weight equal to or greater than 15% compared with the average body weight was also considered to be an endpoint.

### 2.11. Statistical Analysis

The data were analyzed using Prism V.9.0 (GraphPad, San Diego, CA, USA). The statistical analysis was conducted to compare the differences in tumor volume between the therapeutic groups using either a two-way ANOVA or mixed-effects analysis, followed by Tukey’s post-test. A *p*-value of less than 0.05 was considered to be statistically significant.

### 2.12. Ethical Statement

Mouse experiments were performed under a project license (license number 06/2021) granted by the Veterinäramt des Kantons Zürich, Switzerland, in compliance with the Swiss Animal Protection Act (TSchG) and the Swiss Animal Protection Ordinance (TSchV).

## 3. Results

**Figure 1:**
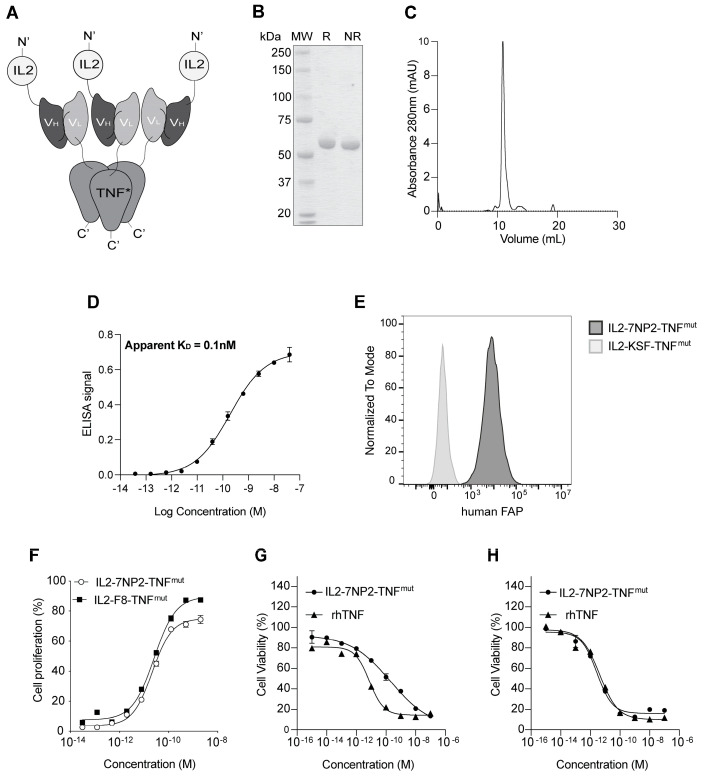
Biochemical characterization of IL2-7NP2-TNF^mut^. (**A**) Schematic representation of the fusion protein assembly in non-covalent homotrimers. (**B**) SDS-PAGE analysis (MW: molecular weight; R: reducing condition; NR: non-reducing condition). (**C**) Size-exclusion chromatography profiles of IL2-7NP2-TNF^mut^ (180 kDa; black line), IgG (150 kDa; segmented line), and SIP (80 kDa; dotted line) were used as controls for the column’s equilibration [25]. (**D**) Titration ELISA on human FAP-coated plate. (**E**) Flow cytometry on SKRC52-hFAP cells. (**F**) IL2 proliferation assay on CTLL2 cells. (**G**) TNF killing assay on SKRC52 wt cells and (**H**) SKRC52-hFAP cells.

The 7NP2 antibody, specific to human FAP [45], in an ScFv format was fused to the wild-type human IL2 at the N-terminus and to a mutated version of human TNF at the C-terminus [33]. The final construct, termed IL2-7NP2-TNF^mut^ (Figure 1A), showed a similar arrangement to the previously described IL2-F8-TNF^mut^ molecule [33,34,47].

The fusion protein was produced in mammalian cells and purified on Protein A resin, exploiting the VH properties of the 7NP2 antibody. In the solution, the protein formed stable non-covalent homotrimers mediated by the assembly of three TNF moieties. The product showed a single band in SDS-PAGE (Figure 1B) corresponding with the monomer and a single peak in gel filtration indicating the trimer (Figure 1C). The affinity of IL2-7NP2-TNF^mut^ for its cognate antigen was measured by ELISA on recombinant human FAP with an estimated K_D_ of 0.1 nM (Figure 1D). The binding to FAP of IL2-7NP2-TNF^mut^ was further confirmed by flow cytometry on SKRC52-hFAP cells compared with the negative control IL2-KSF-TNF^mut^ (Figure 1E; Supplementary Figure S2).

The novel fusion protein was characterized by an intact mass analysis via mass spectrometry. It was found mainly in its non-glycosylated form (86%), whereas a molecular weight shift of + 657 Dalton was found in ~14% of the population. This mass shift corresponded with O-linked glycosylation, particularly HexNAcHexNeuAc (Supplementary Figure S3A). A glycopeptide analysis via a bottom-up approach identified the modification site on a serine of the TNF moiety (Supplementary Figure S3B). We also investigated the denaturation profile by differential scanning fluorimetry, measuring an apparent melting temperature at 44.5 °C (Supplementary Figure S4).

IL2-7NP2-TNF^mut^ retained an intact IL2 activity, as evidenced by an in vitro CTLL2 proliferation assay (Figure 1F). An in vitro killing assay on SKRC52 wt cells revealed a reduction in TNF potency (Figure 1G) due to a single amino acid substitution [33]. Interestingly, the cytotoxic activity of IL2-7NP2-TNF^mut^ was restored when tested on SKRC52-hFAP cells (Figure 1H).

**Figure 2:**
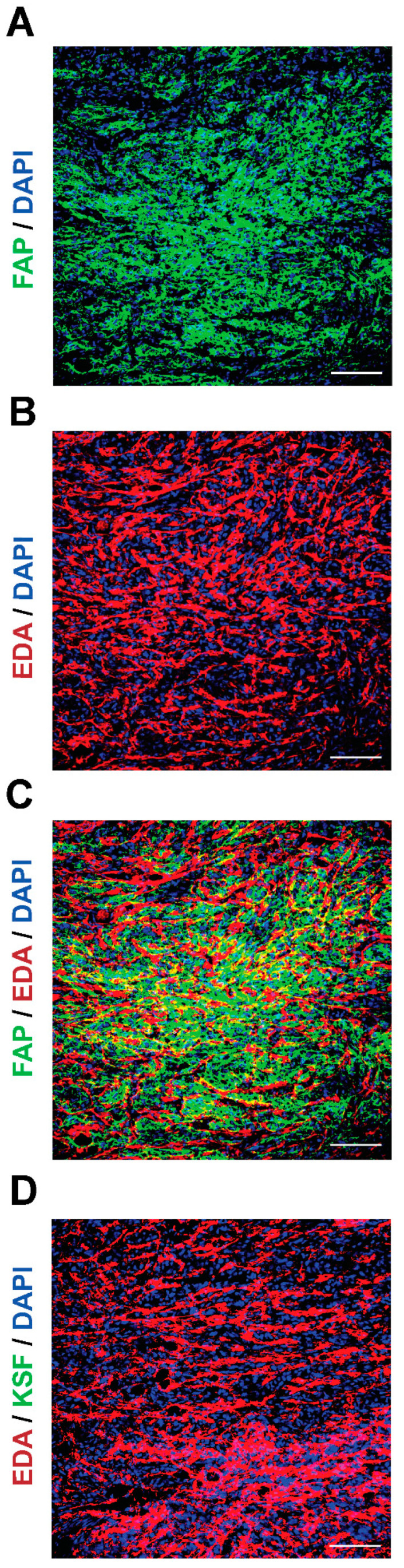
Antigen expression in SKRC52-hFAP tumor sections. Microscopic fluorescence analysis of (**A**) human FAP (green) and (**B**) EDA (red) expressions, and (**C**) merged image. (**D**) Staining with an unspecific antibody (KSF) and EDA (red). Magnification: 20×; scale bars: 100 μm.

Next, we compared the antigen expression patterns in SKRC52-hFAP tumor sections by microscopic fluorescence (Figure 2) using 7NP2 IgG1 [45] and F8 IgG2a [48]. In this model, human FAP expression was restricted on the cell surface (Figure 2A; green staining). At the same time, fibronectin’s extra domain A (EDA) was found in the extracellular matrix (Figure 2B; red staining). When merged (Figure 2C), the two antigens showed a high expression in the whole section. The KSF antibody (specific to an irrelevant antigen) [47] was used as a negative control, and no signal was detected in the green channel (Figure 2D).

**Figure 3:**
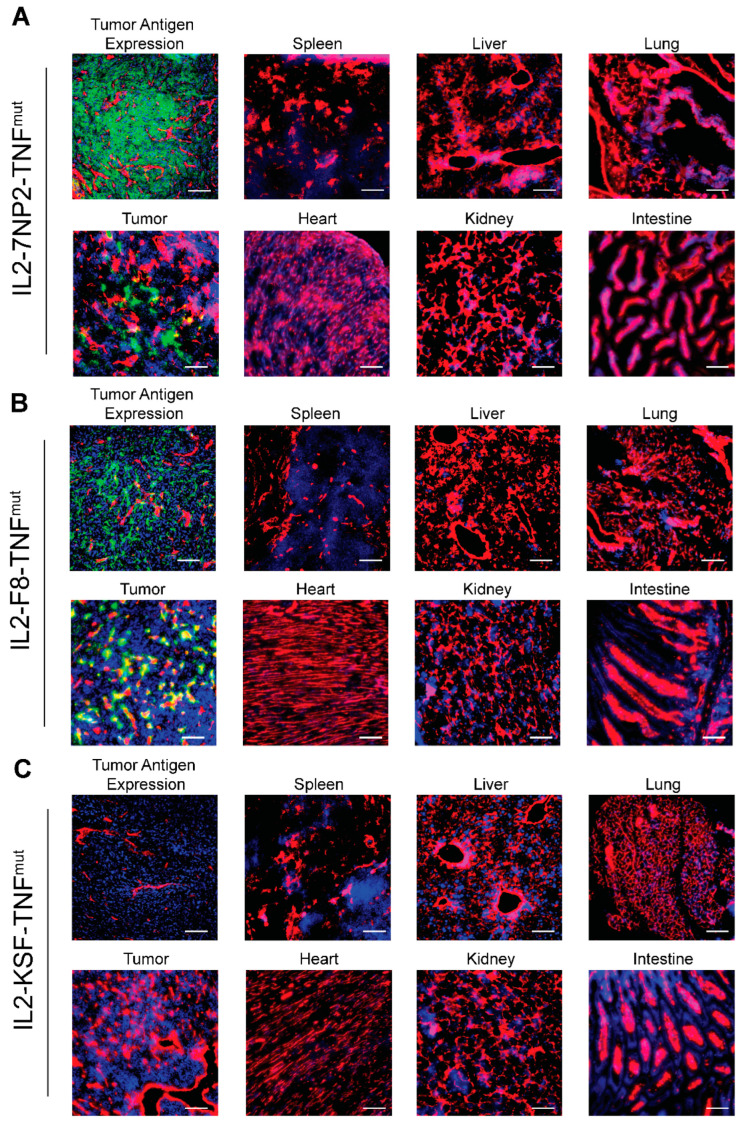
Antigen expression and tumor-targeting properties of IL2-7NP2-TNF^mut^ on SKRC52-hFAP tumor sections. (**A**) Upper left displays human FAP expression detected with IL2-7NP2-TNF^mut^ (green), followed by microscopic fluorescence analysis of organs 24 h after intravenous administration of IL2-7NP2-TNF^mut^. (**B**) Similarly, EDA expression detected with IL2-F8-TNF^mut^ (green) on the upper left and microscopic fluorescence analysis of organs 24 h after intravenous administration of IL2-F8-TNF^mut^. (**C**) Tumor sections stained with negative control (IL2-KSF-TNF^mut^) and detection in organs after intravenous administration. Cryo-sections were stained with anti-IL2 (green), anti-CD31 (red), and DAPI (blue). Magnification: 20×; scale bars: 100 μm.

The in vivo tumor-targeting properties of IL2-7NP2-TNF^mut^ were studied in BALB/C nude mice bearing subcutaneous SKRC52-hFAP tumors. An amount of 60 µg IL2-7NP2-TNF^mut^, IL2-F8-TNF^mut^, and IL2-KSF-TNF^mut^ was injected in the tail vein, and organs were examined after 24 h by immunofluorescence studies. An in vitro analysis of the tumor sections revealed homogenous staining by IL2-7NP2-TNF^mut^, confirming the cellular expression of human FAP similar to the staining observed with 7NP2 in an IgG1 format. Despite the rich presence of a cellular antigen, IL2-7NP2-TNF^mut^ achieved a partial localization in the in vivo setting, failing to uniformly localize in the whole tumor section (Figure 3A). No antibody uptake was detectable in the healthy organs. By contrast, IL2-F8-TNF^mut^ was able to stain EDA in the extracellular matrix and around the blood vessels, and efficiently localize in neoplastic lesions (Figure 3B). The administration of the negative control IL2-KSF-TNF^mut^ did not result in any uptake in the tumor or normal organs (Figure 3C).

**Figure 4:**
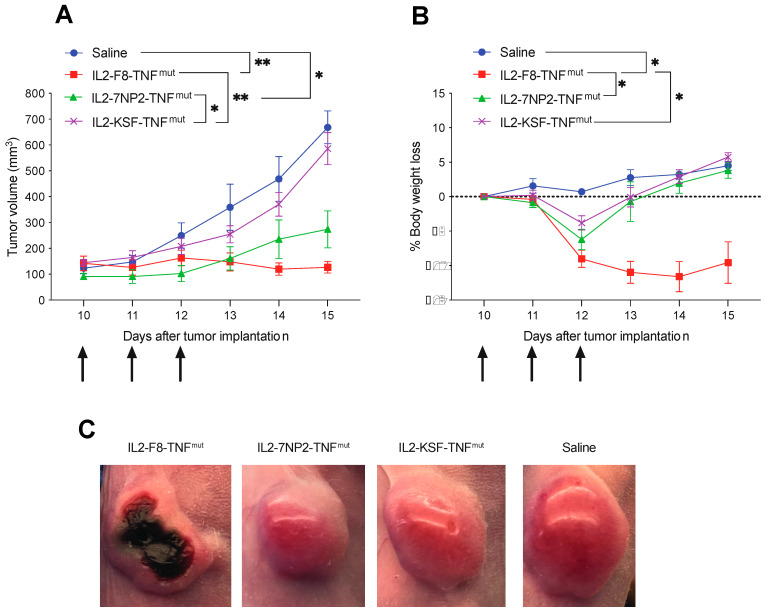
Therapeutic performance of IL2-7NP2-TNF^mut^ compared with IL2-F8-TNF^mut^ in BALB/C nude mice bearing SKRC52-hFAP tumors. Treatments started at day 8 when tumors reached a volume of 70–100 mm^3^; mice were injected intravenously 3 times every 24 h with 30 μg of fusion proteins. As controls, saline and IL2-KSF-TNF^mut^ mice groups were included. (**A**) Results are expressed as tumor volume in mm^3^ ± SEM and (**B**) % mean body weight change ± SEM. For therapy experiments, *n* = 5 mice/group. (* *p* < 0.5; ** *p* < 0.01). (**C**) Tumor macroscopic appearance during treatment: from left to right, example of mice treated by IL2-F8-TNF^mut^, IL2-7NP2-TNF^mut^, IL2-KSF-TNF^mut^, and saline.

The therapeutic activity of IL2-7NP2-TNF^mut^ was compared with IL2-F8-TNF^mut^ and IL2-KSF-TNF^mut^ in mice bearing SKRC52-hFAP tumors (Figure 4A). Mice treated with IL2-F8-TNF^mut^ showed significant tumor growth retardation compared with the mice treated with saline or with the negative control molecule IL2-KSF-TNF^mut^. In this setting, complete responses were not achieved due to the lack of functional T lymphocytes. A modest tumor growth inhibition was observed in the mice treated with IL2-7NP2-TNF^mut^ (*p*-value of 0.0145 at day 15). The body weight profiles showed a transient reduction in all treated groups compared with saline, which was especially pronounced in the IL2-F8-TNF^mut^ group (Figure 4B). At the macroscopic level, after the first intravenous injection, a formation of tumor necrosis was observed in the mice treated with IL2-F8-TNF^mut^, but not in the mice treated with saline, IL2-7NP2-TNF^mut^, or IL2-KSF-TNF^mut^ (Figure 4C).

## 4. Discussion

In this work, we have reported a new dual-cytokine antibody fusion protein that combined the anti-FAP antibody fragment 7NP2 [45] with IL2 and a depotentiated version of TNF.

The incorporation of TNF in anticancer dual-cytokine therapeutics is particularly attractive for several reasons. On the one hand, TNF subunits assemble in stable non-covalent homotrimers, leading to an increased binding avidity of the multivalent antibody fusion protein. On the other hand, TNF has unique vasoactive functions, altering the blood flow and permeability of vessels, which favors the fast extravasation and tumor uptake of other drugs. Moreover, TNF stimulates the immune system, synergizes with other payloads, and promotes hemorrhagic necrosis of the neoplastic mass [49,50,51,52]. A number of TNF fusion proteins, capable of a preferential localization at the tumor site, are currently being investigated at the clinical level. NGR-TNF and L19-TNF are examples of successful immunoconjugates targeting tumor vasculature undergoing phase II/III clinical trials for mesotheliomas and melanomas [20,53].

Previous studies on multifunctional cytokine–antibody fusion proteins have shown that therapeutic efficacy is strongly influenced by: (i) the biology of cytokines; (ii) the chosen cytokine payloads; and (iii) the molecular design [23,24,25,26,27,28,33,34,48]. To combine IL2 and TNF in a single product, it is essential to consider the maximal tolerated doses (MTDs) of both payloads in vivo. The MTD of targeted TNF is 5–10 times lower than that of targeted IL2 in mice and cancer patients. When the biological activity of the cytokine payload is not in a comparable molar range, it is challenging to fully exploit the therapeutic or synergistic effect. For this reason, our group depotentiated the TNF moiety by introducing a single-point mutation (TNF^mut^), shifting its potency closer to the one of IL2 [33]. In principle, the introduction of amino acid substitutions could be immunogenic at the clinical level. Various classes of therapeutic proteins (e.g., engineered insulins) can be safely administered to patients for years without mounting neutralizing antibodies [54].

We studied the in vivo activity of IL2-7NP2-TNF^mut^ (directed to tumor cells) and IL2-F8-TNF^mut^ (directed to the tumor neovasculature and stroma) in a xenograft model of renal cell carcinomas overexpressing human FAP on the cell surface [33,34]. Both fusion proteins showed favorable tumor-homing properties 24 h after an intravenous injection. Surprisingly, only mice treated with IL2-F8-TNF^mut^ benefited from the treatment, and distinctive tumor necrosis was visible at the macroscopic level.

In our hands, targeting an antigen expressed on the cell membrane of transfected tumor cells has not led to the same therapeutic outcome as targeting a stromal antigen. Nevertheless, more examples need to be studied to learn whether this limited observation may represent a general principle for the design of antibody–cytokine fusion proteins.

In summary, IL2-7NP2-TNF^mut^ showed encouraging tumor-homing properties in lesions expressing cell membrane-bound FAP, a target that has been extensively validated by nuclear medicine procedures as an excellent accessible marker of various types of solid malignancies [44,55,56,57]. It remains to be seen whether ECM components (such as the splice variants of fibronectin) or cellular antigens may represent equivalent targets for pharmacodelivery applications, or whether one class of targets may outperform the other as a result of better accessibility and a higher proximity to the tumor endothelium.

## Data Availability

All related data and methods are presented in this paper and Supplementary Materials. Additional inquiries should be addressed to the corresponding author.

## Supplementary Materials

The following supporting information can be downloaded at: https://www.mdpi.com/article/10.3390/antib12020029/s1, Supplementary Materials: Supplementary methods; Figure S1: Amino acid sequence of IL2-7NP2-TNF^mut^. Figure S2: Gating strategy for Flow Cytometry Analysis on SKRC52-hFAP cells. From left to right: ungated cell population, single cell population, live cells and antigen positive cells.; Figure S3: Mass Spectrometry analysis of IL2-7NP2-TNFmut. A) Intact Mass Analysis reporting a putative O-glycosylation variant (+657 Da). B) Glycopeptide analysis confirming the O-glycosylation and identifying its position on the TNFmut moiety; Figure S4: Differential Scanning Fluorimetry of IL2-7NP2-TNF^mut^. The denaturing profile of the fusion protein displays a clear transition at 44.5 °C.
