# Supplementary material for: An Antibody Targeting Fibroblast Activation Protein Simultaneously Fused to Interleukin-2 and Tumor Necrosis Factor Selectively Localizes to Neoplastic Lesions
Source: Antibodies (Basel). 2023 Apr 14;12(2):29. doi: 10.3390/antib12020029 (PMC10123652; doi:10.3390/antib12020029)
Supplement: Supplementary file 1 [file antibodies-12-00029-s001.zip › antibodies-2243906-supplementary.pdf]

Supplementary Information

APTSSSTKKTQLQLEHLLLDLQMILNGINNYKNPKLTRMLTFKFYMPKKATELKHLQCLEEELKPLEEVLNLAQSKNFHLRPRDLISNINVIVLELKGSE
TTFMCEYADETATIVEFLNRWITFCQSIISTLTGGGGSGGGGSGGGGSKEVQLLESGGGLVQPGGSLRLSCAASGFTFSSYAMSWVRQAPGKGLEWVSAI
GSVGGPTYYADSVKGRFTISRDNSKNTLYLQMNSLRAEDTAVYYCAKRLAWFDYWGQGTLVTVSSGGGGSGGGGSGGGGSKEIVLTQSPGTLSLSPGERA
TLSCRASQSVSSSYLAWYQQKPGQAPRLLIYGASSRATGIPDRFSGSGSGTDFTLTISRLEPEDFAVYYCQQSGKGPLTFGQGTKVEIKGGGGSGGGGSG
GGGSKVRSSSRTPSDKPVAHVVANPQAEGQLQWLNRAANALLANGVELRDNQLVVPSEGLYLIYSQVLFKGQGCPSTHVLLTHTISRIAVSYQTKVNLLS
AIKSPCQRETPEGAEAKPWYEPIYLGGVFQLEKGDRLSAEINRPDYLDFAESGQVYFGIIAL

Supplementary Figure S1 Amino acid sequence of IL2-7NP2-TNF^{mut}

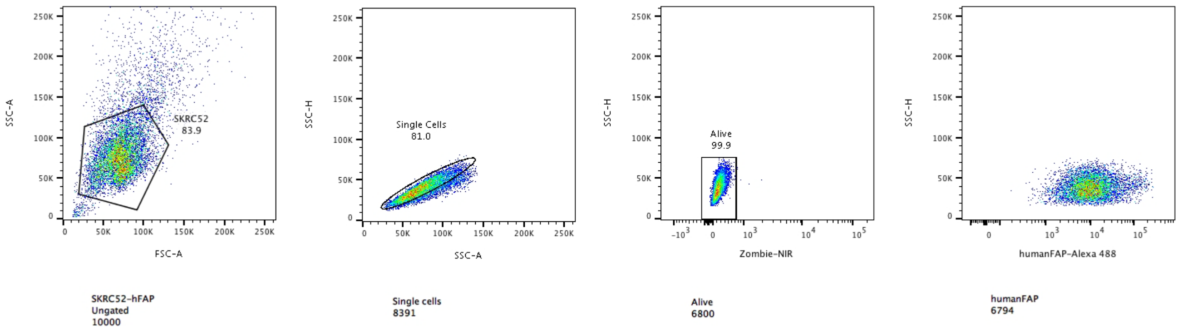

Supplementary Figure S2 Gating strategy for Flow Cytometry Analysis on SKRC52-hFAP cells. From left to right: ungated cell population, single cell population, live cells and antigen positive cells.

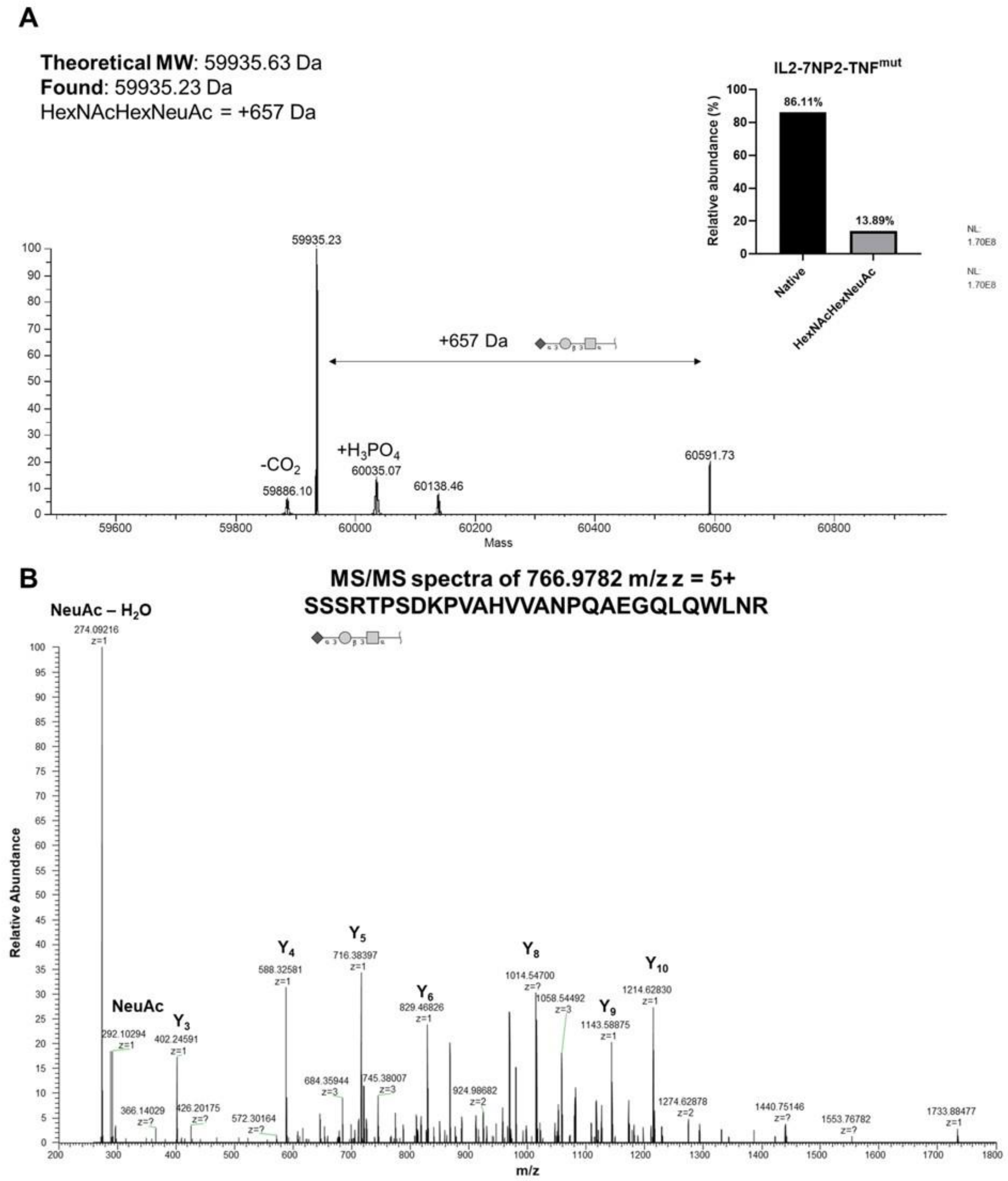

Supplementary Figure S3 Mass Spectrometry analysis of IL2-7NP2-TNF^{mut}. A) Intact Mass Analysis reporting a putative O-glycosylation variant (+657 Da). B) Glycopeptide analysis confirming the O-glycosylation and identifying its position on the TNF^{mut} moiety.

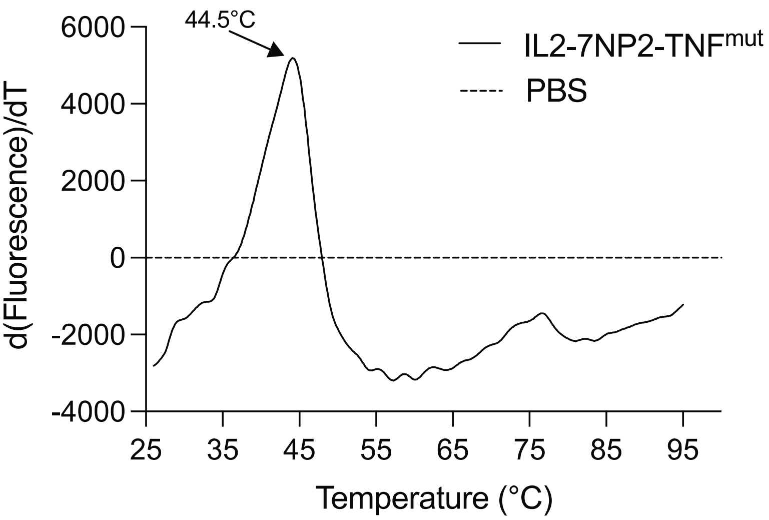

Supplementary Figure S4 Differential Scanning Fluorimetry of IL2-7NP2-TNF^{mut}. The denaturing profile of the fusion protein displays a clear transition at 44.5°C.

Supplementary methods

Intact Mass Analysis

60 µg of protein were desalted using C18 Micro Spin Columns (Harvard Apparatus). The eluate from C18 purification was adjusted to 48 % CH_3CN / 0.2 % HCOOH and then directly injected into a Q Exactive mass spectrometer (Thermo Scientific) equipped with an ESI Ion Max source (Thermo Fisher). Source parameters were set as: polarity positive ion mode; resolution (FWHM at 200 m/z) 17500; microscan 10; S-lens RF level 90; spray voltage 3.5 kV; scan range 500-3500 m/z, in-source CID offset voltage of 80 eV.

Glycopeptide analysis

7.5 µg of sample were diluted to a final volume of 200 µL with an aqueous solution containing Urea 1 M, 50 mM Tris-HCl, 1 mM $CaCl_2$ pH 8.0. The protein was reduced with TCEP for 15 min at RT followed by 30 min at 65 ˚C and alkylated with Iodoacetamide for 30 mins in the dark. Digestion was achieved with trypsin (enzyme-protein ratio 1:50) at 37 ˚C overnight. After digestion, the sample was acidified with 10% formic acid and then subjected to C18 purification and desalting (Macro Spin Columns, Harvard Apparatus). The purified sample was dried under vacuum and resuspended in 30 µL of an aqueous solution containing 3% acetonitrile and 0.1% formic acid. 2 µL of the sample was then subjected to nanoHPLC-HRMS analysis, consisting of an Orbitrap Q-Exactive mass spectrometer coupled to an EASY nanoLC 1000 system via a Nano Flex ion source. Chromatographic separation was carried out at room temperature on an Acclaim PepMap RSLC column (50 µm x 15 cm, particle size 2 µm, pore size, 100 Å), using 30 min linear gradient with 5-35% solvent B (0.1% formic acid in acetonitrile) at a flow rate of 300 nL/min. Ionization was carried out in positive ion mode, with 2 kV of spray voltage, 250 °C of capillary temperature, 60 S-lens RF level. The mass spectrometer was working in a data-dependent mode. MS1 scan range was set from 350 to 1650 m/z, the 10 most abundant peptides were subjected to HCD fragmentation with NCE of 25. A dynamic exclusion was set at 20 seconds. The raw file was processed with Proteome Discoverer 2.5 (Thermo Fisher) for qualitative analysis. Database searches were performed with Sequest as a search engine using a FASTA file containing the aminoacidic protein sequence, Carbamidomethylation of cysteines was set as a fixed modification, O-glycosylations (i.e., Hex, HexNAc, HexHexNAc, HexHexNAcNeuAc, HexHexNAc(NeuAc)2) were set as dynamic modification on serine and threonine, and trypsin was set as cleavage specificity allowing a maximum of 2 missed cleavages.
